# First Taxonomically Curated DNA Barcode Reference Library for Polish Myriapods Fills an Important Knowledge Gap for Soil Invertebrates in Central Europe, Part I: Millipedes (Myriapoda: Diplopoda)

**DOI:** 10.1002/ece3.74268

**Published:** 2026-09-03

**Authors:** Jakub Bienias, Łukasz Trębicki, Gabriela Karlik, Michał Grabowski

**Affiliations:** ^1^ Faculty of Biology and Environmental Protection, Department of Hydrobiology and Invertebrate Zoology University of Lodz Lodz Poland; ^2^ Doctoral School of Exact and Natural Sciences, Faculty of Biology and Environmental Protection University of Lodz Lodz Poland

**Keywords:** cryptic diversity, Diplopoda, DNA barcoding, reference library

## Abstract

DNA barcoding is a highly effective tool for species identification, complementing taxonomic and phylogenetic studies while also playing a key role in biomonitoring and uncovering cryptic diversity. Techniques such as DNA barcoding, metabarcoding and environmental DNA (eDNA) analysis are widely applied in biodiversity conservation, tracking invasive species and studying trophic interactions. To carry out this type of research effectively, a robust DNA barcode library is essential, built upon high‐quality genetic sequences and accurate taxonomic identification. Here, we present the results of a large‐scale DNA barcoding study of Polish diplopod species (Myriapoda: Diplopoda), providing an up‐to‐date checklist of species recorded in Poland and a reference DNA barcode library. We deliver data on 981 individuals identified to species level, of which 468 have COI sequence data. 58 out of 93 species recorded for Poland were successfully barcoded, representing 62% of the national fauna. Additionally, we explore future research directions, potential cryptic diversity and shifts in the national fauna, including newly recorded species for Poland—*Melogona broelemanni* (Verhoeff, 1897). Our findings highlight the significance of ongoing monitoring and molecular methods in tracking biodiversity changes.

## Graphical abstract

DNA barcoding enabled the creation of the first large, taxonomically verified barcode library for Polish millipedes, covering 981 individuals and 58 species (62% of the national fauna), with 468 COI sequences. The study reveals potential cryptic diversity, shifts in the Polish diplopod fauna and documents Melogona broelemanni as a species newly recorded for Poland. These results highlight the importance of continuous monitoring and molecular tools in tracking biodiversity change.

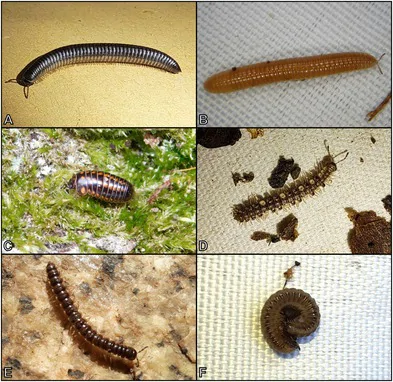

## 1 Introduction

Millipedes (Diplopoda) are a species‐rich subgroup of arthropods, comprising more than 12,000 described species distributed across all continents except Antarctica (Golovatch and Kime 2009; Brewer et al. 2012). They inhabit almost all terrestrial habitats and rely on high humidity and abundance of shelters such as leaf litter, stones or dead wood (Golovatch and Kime 2009). Some millipede species are known to inhabit caves; many are troglobionts, especially in orders like Chordeumatida and Polydesmida (Shear and Krejca 2019). Among cavernicolous species, rare cases of semi‐aquatic or hygropetric lifestyles have also been reported (Enghoff et al. 1997; Antić and Akkari 2023). Plenty of species have spread to other countries and continents as alien and invasive species, often occurring in urbanised habitats such as city parks, gardens or hothouses (Stoev et al. 2010).

Most millipedes are typical forest‐floor detritivores (Golovatch and Kime 2009), and along with earthworms, they are among the most important groups of litter‐feeding macroinvertebrates in temperate regions (Hättenschwiler and Gasser 2005). In forest soils, their population density can exceed 1000 individuals per square metre and they are believed to process up to 10%–15% of the annual leaf litter in temperate forests (Golovatch and Kime 2009). Thus, they facilitate faster decomposition and nutrient recycling in forest ecosystems (Stašiov et al. 2021). Only a few millipedes of the order Callipodida exhibit predatory behaviour, such as the Balkan species *Apfelbeckia insculpta* (Koch 1867), which has been reported to feed on earthworms, flies and even predators such as spiders and centipedes (Stoev and Enghoff 2008). Few cavernicolous millipedes exhibit unique modifications of mouthparts due to a filtering diet (Antić and Akkari 2023).

Most millipede species use a chemical defence system to deter predators, including substances such as hydrogen cyanide, phenols, benzoquinones, or glomerin, depending on the taxonomic group (Shear 2015). Despite these defences, millipedes are preyed upon by many animals, including large mammals such as the European wild boar, birds and insects like beetles (Schley and Roper 2003; Brunke et al. 2009; Aradis et al. 2019). Most predators feed only occasionally on millipedes; however, some arthropods, such as members of the hemipteran subfamily Ectrichodiinae, are specialised millipede predators (Forthman and Weirauch 2012). Millipedes are also targeted by parasites, such as phorid flies of the genus *Myriophora*, which use millipede defensive secretions as kairomones to detect their hosts (Hash et al. 2017).

Given that expertise in millipede taxonomy has historically been concentrated among only a handful of specialists, collection gaps have persisted in certain regions (Sierwald and Bond 2007). Additionally, much of taxonomy has focused on analysing gonopods (male sperm‐transfer appendages). However, not all millipede lineages have these structures: Polyxenida completely lacks them, whereas members of Pentazonia possess telopods instead. In general, groups in which these are unreliable diagnostic features (like Colobognatha) are often understudied (Brewer et al. 2012). This also implies that individuals lacking this character, such as juveniles and females, are often very hard or impossible to identify to species level (Wesener et al. 2016; Bienias et al. 2026).

This is one of the shortcomings that can be remedied by DNA barcoding, a method of molecular species identification that has gained increasing importance over the last 20 years (Hebert, Cywiska, et al. 2003; Hebert, Ratnasingham, and de Waard 2003). In animals, DNA barcoding typically relies on a standardised 658‐base‐pair 5′ fragment of the mitochondrial cytochrome c oxidase subunit I (COI) gene as the primary diagnostic marker (Hebert, Cywiska, et al. 2003; Hebert, Ratnasingham, and de Waard 2003). The effectiveness of DNA barcoding is fundamentally dependent on the public availability of robust, expertly curated reference libraries, such as those deposited in the Barcode of Life Data System (BOLD), which can provide the necessary framework for matching unknown sequences to known species (Ratnasingham and Hebert 2007, 2013). In recent years, the field has increasingly transitioned from traditional Sanger sequencing toward High‐Throughput Sequencing (HTS) platforms. Specifically, Oxford Nanopore Technologies now enables the rapid, cost‐effective generation of large‐scale genomic data, facilitating the identification of thousands of specimens in a single workflow (Srivathsan et al. 2021; Baloğlu et al. 2021). Moreover, DNA barcoding has become a cornerstone of integrative taxonomy, offering a powerful tool for delineating evolutionary lineages and uncovering cryptic diversity in groups that are genetically distinct but morphologically indistinguishable, as well as for biomonitoring, detecting non‐native species and understanding biological invasions (DeSalle and Goldstein 2019; Comtet et al. 2015; Hering et al. 2018; Hollingsworth et al. 2026). In the study of myriapods, the use of DNA barcodes and other molecular markers has often led to remarkable discoveries, including integrative descriptions of species, clarification of relationships between problematic taxa, the recording of previously overlooked species, as well as insights into intraspecific variation or polyphyly (Spelda et al. 2011; Wesener 2015; Wesener and Conrad 2016; Moritz and Wesener 2017; Reip and Wesener 2018). Such an integrative approach is especially useful for groups with highly chaotic and outdated taxonomy, as seen in the genus *Glomeris* (Wesener 2015). DNA barcoding can also be used to identify female and juvenile myriapods, whose identification is often extremely difficult or even impossible based on morphology (Wesener et al. 2016; Bienias et al. 2026).

**FIGURE 1:**
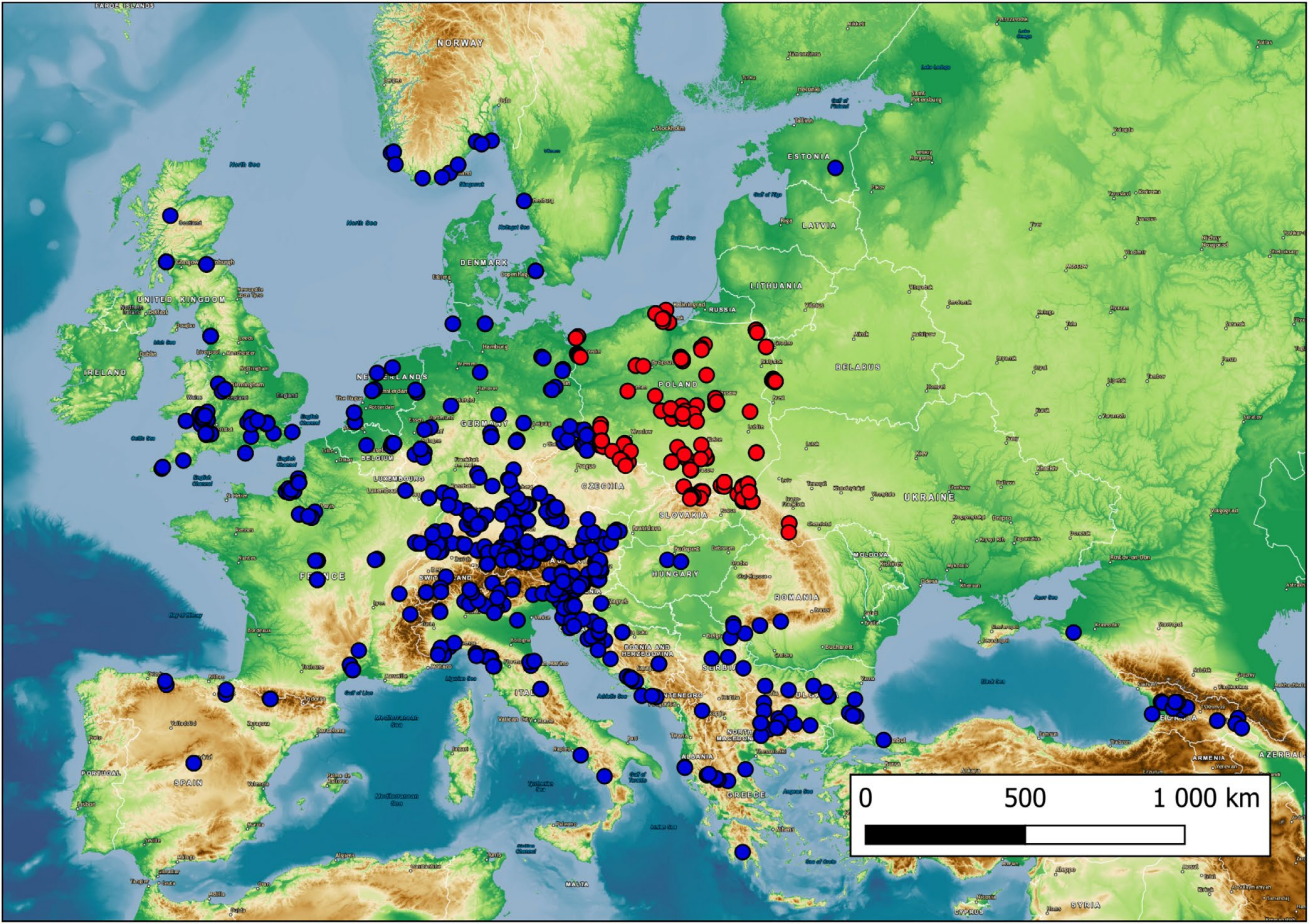
Map of localities from which DNA‐barcodes of European millipedes are stored in BOLD. Blue dots represent public sequences obtained from the BOLD System, whereas red dots represent sequences obtained in this study.

Large‐scale studies on diplopods using molecular markers have been successfully conducted in the neighbouring Germany (Spelda et al. 2011; Wesener 2015), as well as in other European (Wilbrandt et al. 2015; Wesener and Conrad 2016) and non‐European countries (Moritz and Wesener 2017; Pimvichai et al. 2018; Likhitrakarn et al. 2024; Fiemapong et al. 2025; Li et al. 2025). Nevertheless, many regions of the world, including some parts of Europe (Figure 1), still lack molecular data on millipedes.

Despite the rich diversity of Polish diplopods, the lack of molecular data, such as COI barcodes, hinders the resolution of taxonomic uncertainties within the group, including the accurate identification of potential cryptic species. In fact, until now, there have been almost no studies, with the only exceptions being a few papers on a few millipede species, *Chondrodesmus riparius* Carl, 1914 (Bienias et al. 2021), *Polydesmus angustus* Latzel, 1884b (Bienias et al. 2025) and *Cylindroiulus vulnerarius* (Berlese 1888) (Bienias et al. 2026), which include DNA sequence data.

The primary objective of our study is to establish the first comprehensive DNA barcode reference library for Polish Diplopoda, effectively bridging a major geographical gap in knowledge of the molecular diversity of European myriapods. This work is highly original as it moves beyond traditional morphological records, providing the first large‐scale application of COI barcoding to the millipede fauna of Poland, a region that has remained largely uncharacterized molecularly compared to neighbouring countries. Furthermore, we provide a critical update to the national checklist, documenting also, for the first time, the presence of the chordeumatidian species *Melogona broelemanni* (Verhoeff 1897b) in Poland. By integrating these molecular data with an exhaustive faunistic review, our study offers a novel, baseline framework for future biodiversity monitoring and evolutionary studies in Central Europe.

## 2 Material and Methods

**FIGURE 2:**
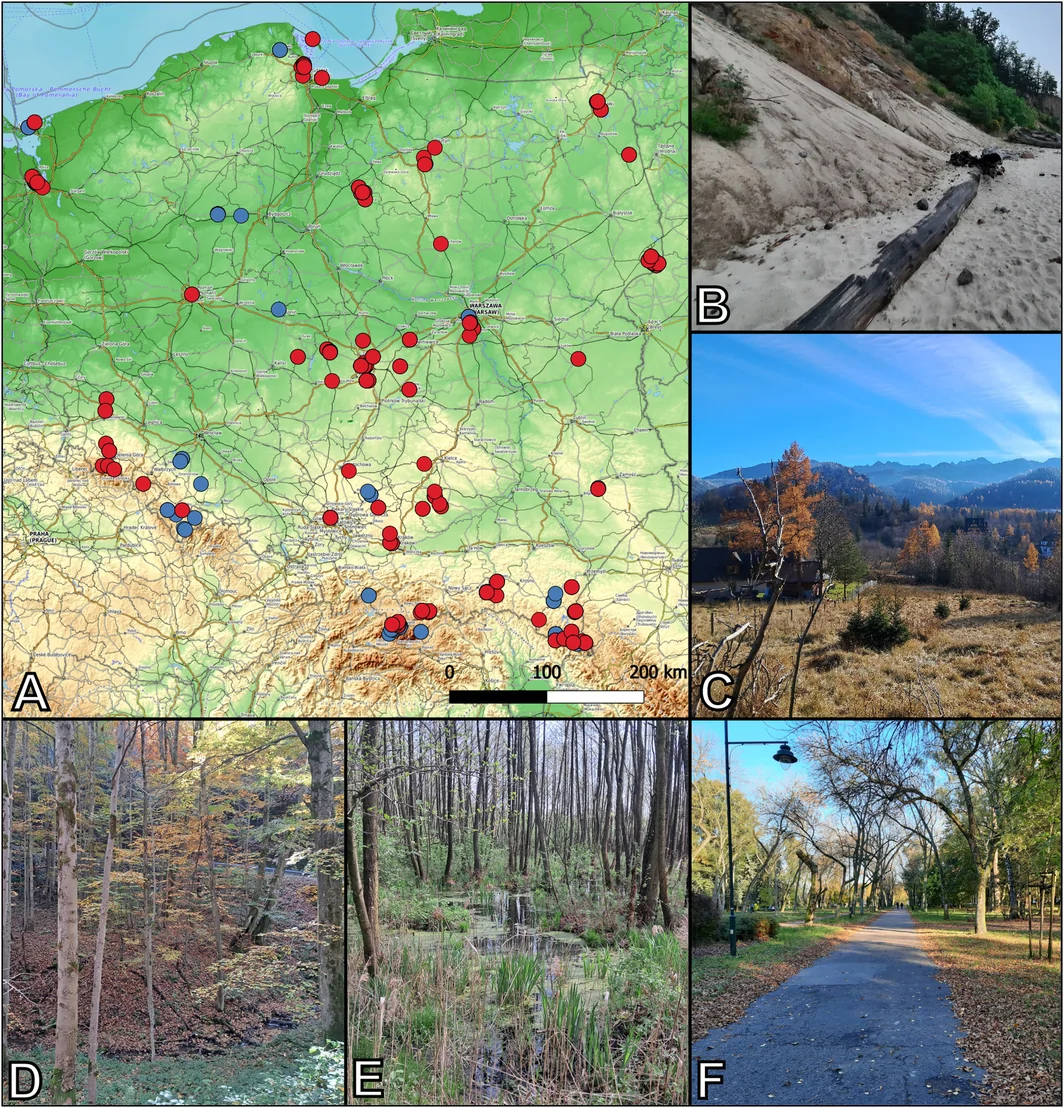
Map of the localities sampled for this study (A). Red dots represent material collected recently or for the purposes of this study, while blue dots represent historical material borrowed from the MIIZ PAN. Examples of the sampled habitats are shown in panels (B–F), Baltic shore near Gdynia (B), a hill in Zakopane (C), beech forest in the Bieszczady Mountains (D), the Lipickie Błota wetland (E), ‘Zdrowie’ city park in Łódź (F).

Millipedes were sampled across the country, mostly using qualitative methods such as litter sifting and hand collecting. The collected material comes from 96 localities in total (Figure 2). Most samples were collected recently, specifically for this study (61 localities) between 2023 and 2025; however, material from previous years was also included. Historical material from 35 localities was incorporated as well. This material was borrowed from the Museum and Institute of Zoology of the Polish Academy of Sciences (MIIZ PAN) and consisted mostly of species that were not found during the field trips, are rare, or whose taxonomic validity is questionable, as in the case of *Glomeris mnischechi* Nowicki, 1870 (see Haľková et al. 2021). Most samples originate from Poland, with only a few from Slovakia and Ukraine; the latter were used for comparative purposes. Almost all specimens were initially identified using Stojałowska's (1961) key to Polish diplopod species, and the identifications were confirmed subsequently using other literature on Central and Eastern European diplopods (Lang 1954; Kocourek et al. 2017; Giurginca 2021), and, in some cases, original species descriptions (Jawłowski 1930; Nowicki 1870).

### 2.1 DNA Isolation

Total genomic DNA was extracted from body segments or legs using protocols tailored to specimen age and preservation conditions. Freshly collected material was preserved in 96% ethanol and DNA was isolated using a Chelex‐based protocol as described by Casquet et al. (2012).

From older specimens preserved in 75% ethanol, we extracted DNA according to a modified magnetic bead‐based extraction protocol, originally proposed by Tin et al. (2014), suitable for degraded DNA, which is usually present in historical material (Derkarabetian et al. 2019). Two key modifications were introduced: (1) guanidine isothiocyanate in the lysis buffer was replaced with guanidine thiocyanate; and (2) the original G‐Biosciences magnetic beads were substituted with MagAttract Suspension G beads (Qiagen, Hilden, Germany), supplied as part of the MagAttract HMW DNA Kit. All extractions using the magnetic bead protocol were performed manually on a magnetic stand. DNA extractions of museum specimens were performed according to Hall et al. (2023).

### 2.2 PCR Amplification and Sequencing

PCR amplification of the mitochondrial cytochrome c oxidase subunit I (COI) barcode region was performed for all samples for which DNA quality allowed successful amplification. For freshly collected specimens, sequencing was carried out using two complementary approaches. First, standard Sanger sequencing was applied for high‐quality DNA extracts. Second, a subset of samples was sequenced using Oxford Nanopore Technologies (ONT) on the MinION platform, enabling efficient multiplexed amplicon processing.

### 2.3 PCR Amplification and Sequencing of Fresh Material

For the purpose of Sanger sequencing, the COI barcode fragment was amplified using the primer pair LCO1490‐JJ and HCO2198‐JJ following Astrin and Stüben (2008). PCR conditions and subsequent purification of amplicons were performed according to the protocol described by Querner et al. (2022). The sequencing of purified PCR products using the forward primer was outsourced to Macrogen Europe BV.

DNA barcoding using the Oxford Nanopore Technologies (ONT) platform was performed as described by Srivathsan et al. (2021) with minor modifications. For ONT, the COI barcode fragment was amplified using the primer pair LCO1490‐JJ/HCO2198‐JJ (Astrin and Stüben 2008), each tagged with a unique 9 bp index sequence on both forward and reverse primers for individual specimens. This dual‐indexing strategy enabled unambiguous assignment of reads during demultiplexing. The primer tags were designed using Barcode Generator (Comai and Howell 2012), following the approach of Srivathsan et al. (2019).

PCR reactions were carried out in a total volume of 11 μL, containing 6.5 μL DreamTaq polymerase (Thermo Fisher Scientific), 1 μL (5 μM) of each indexed primer (LCO1490‐JJ and HCO2198‐JJ), 3.5 μL ddH_2_O and 1 μL DNA extract. The thermal cycling profile consisted of an initial denaturation at 95°C for 5 min; five cycles of 95°C for 50 s, 45°C for 50 s and 72°C for 60 s; followed by 35 cycles of 95°C for 50 s, 51°C for 50 s and 72°C for 60 s; with a final extension at 72°C for 2 min.

PCR success rate was verified by electrophoresis on a 1% agarose gel and visualisation of amplicons in UV light. Subsequently, 5 μL of each PCR product was pooled into a single 1.5 mL tube. The pooled amplicons were purified using Sera‐Mag Select beads (Cytiva Life Sciences), and purification success was confirmed again on a 1% agarose gel. DNA concentration of the final pool was measured using a Qubit 4 Fluorometer with the Qubit dsDNA HS Assay Kit (Invitrogen, Thermo Fisher Scientific).

Sequencing of the COI barcode region was performed using the Oxford Nanopore Technologies (ONT) MinION platform, following the protocol described by Srivathsan et al. (2021). Libraries were prepared using the Oxford Nanopore Ligation Sequencing Kit V14 (SQK‐LSK114) according to the standard ONT amplicon sequencing protocol. The protocol aligns with the standard DNA barcoding workflow established at the Department of Invertebrate Zoology and Hydrobiology, University of Lodz (e.g., Królikowska et al. 2024; Bienias et al. 2025), as well as by the PolBOL initiative (polbol.uni.lodz.pl). Basecalling was performed using Dorado v1.4.0 (https://github.com/nanoporetech/dorado) employing the high‐accuracy model dna_r10.4.1_e8.2_400bps_sup@v4.3.0 on the Athena high‐performance computing infrastructure (PLGrid; https://www.plgrid.pl; accessed 4 April 2024). Raw data (FAST5 format) were subsequently demultiplexed and processed into consensus sequences using ONTBarcoder 2 (Srivathsan et al. 2021). The resulting barcodes were validated by comparison against the BOLD Systems database (id.bold) to ensure taxonomic accuracy.

### 2.4 PCR Amplification and Sequencing of Museum Material

In contrast, museum specimens were processed directly from DNA extracts without prior PCR amplification. These samples were subjected to low‐coverage whole‐genome shotgun sequencing (genome skimming), from which mitochondrial sequences, including the COI barcode region, were subsequently recovered bioinformatically (details not included here).

#### 2.4.1 Genome Skimming and Library Preparation

Libraries were prepared at the Natural History Museum London (NHM), using the Santa Cruz Reaction (SCR) protocol (Kapp et al. 2021) with modifications described by Nguyen et al. (2023). Reaction volumes were reduced by 50% relative to the original protocol, with additional minor modifications detailed in Price et al. (2025).

Libraries were pooled equimolarly, with four 96‐well plates combined per pool, and sequenced on an Illumina NovaSeq 6000 platform (25B configuration) at the SNP and SEQ Technology Platform.

#### 2.4.2 COI Barcode Recovery

COI barcodes were recovered with BeeGees (Parsons et al. 2026, accepted), a Snakemake pipeline running MGE (Brasseur et al. 2023) and Exonerate (Slater and Birney 2005). BeeGees was run using Gene Fetch with ‘taxid’ input, retrieving COI protein sequences (> 500 aa) for use as sample‐specific pseudo‐references. Six MGE parameter combinations were used (*r* = 1, 1.3, 1.5; *s* = 50, 100). Downsampling was disabled so that all reads were processed.

The *fasta_cleaner* module was applied with default thresholds (≥ 50% consensus agreement, ≥ 95% removal of sequences matching human COI, ≤ 10% absolute AT‐content difference, and statistical outlier removal), excluding optional reference‐based filtering. Pre‐processing produced up to 24 barcode consensus sequences per sample, which were subjected to structural validation (COI‐5P HMM, genetic code 5) and taxonomic validation using BOLDistilled (July 2025; Prosser et al. 2025), requiring ≥ 80% identity over ≥ 100 bp for at least family‐level identification.

#### 2.4.3 Computational Resources

Analyses at the Natural History Museum were performed on the UK Crop Diversity Bioinformatics HPC (Percival‐Alwyn et al. 2025).

### 2.5 Sequence Processing, Alignment and Data Repository

The obtained sequences were aligned in Geneious v10.2.6 (Biomatters Ltd.) following Kearse et al. (2012) and using the MUSCLE algorithm (Edgar 2004) with default parameters. The resulting alignment was manually inspected, trimmed to remove primer sequences and checked for stop codons, frameshifts and ambiguous base calls, including double peaks in Sanger sequencing chromatograms. All sequences were validated by BLAST searches against the Barcode of Life Data System (BOLD) and the NCBI database to confirm taxonomic identity and exclude potential contamination.

All DNA barcode sequences were deposited in BOLD (https://www.boldsystems.org/) (Ratnasingham and Hebert 2007) and in GenBank (https://www.ncbi.nlm.nih.gov/), together with associated voucher data, taxonomic identifications and specimen images. The complete dataset is publicly available via BOLD (dataset DOI: https://doi.org/10.5883/DS‐PDBL) and GenBank (accession numbers: PZ807765—PZ808217).

### 2.6 Dataset Assembly and Filtering

We analysed a dataset of 461 COI sequences representing 12 Diplopoda families occurring in Poland. Sequences were aligned prior to analysis, and only specimens with valid process identifiers (processID) and assigned family‐level taxonomy were retained. All sequence identifiers were standardised to BOLD process IDs to ensure unambiguous linkage between sequence data and taxonomic metadata.

### 2.7 BIN Assignment

Sequences were clustered into molecular operational taxonomic units using the Barcode Index Number (BIN) system implemented in BOLD Systems (Ratnasingham and Hebert 2013). It automatically assigns COI sequences to BINs within the BOLD platform, employing a three‐step online pipeline to algorithmically cluster sequences into operational taxonomic units (OTUs) based on a refined distance threshold and global connectivity (Ratnasingham and Hebert 2013). For individuals assigned to the same BIN are generally expected to correspond to a single species as delineated by traditional taxonomy (Hausmann et al. 2013); the system effectively serves as a standardised, database‐informed proxy for species. Furthermore, we performed a ‘BIN Discordance’ analysis to identify potential taxonomic inconsistencies, specifically revealing instances where species clusters either shared a single BIN or were split across multiple BINs. In cases of disagreement between morphological identification and genetic clustering, ambiguous specimens were re‐examined using an integrative taxonomic framework (Riedel et al. 2013).

### 2.8 Family‐Level Barcode Gap Analysis and Threshold Optimisation

To evaluate the presence of a barcode gap at the species level, pairwise genetic distances were partitioned into intraspecific and interspecific comparisons. Intraspecific distances were calculated from all pairwise comparisons among sequences assigned to the same species, whereas interspecific distances were obtained from comparisons among different species within the analysed dataset.

Genetic distances were calculated using the Kimura 2‐parameter (K2P) model (Kimura 1980), which accounts for unequal rates of transitions and transversions and is widely applied in DNA barcoding studies (e.g., Spelda et al. 2011; Królikowska et al. 2024; Bienias et al. 2025).

The analysis was first performed for the complete Diplopoda dataset (454 sequences representing 58 species). To assess barcode gap patterns within well‐sampled taxonomic groups, the same analysis was repeated separately for the five most extensively represented families: Julidae, Polydesmidae, Glomeridae, Blaniulidae and Mastigophorophyllidae.

The distribution of intraspecific and interspecific distances was visualised using boxplots to assess the extent of barcode gap separation, following standard DNA barcoding frameworks (Hebert, Cywiska, et al. 2003; Hebert, Ratnasingham, and de Waard 2003).

For each dataset, an optimal genetic distance threshold (OT) was estimated to maximise discrimination between intraspecific and interspecific comparisons. Threshold values were evaluated across all observed K2P distances, and pairwise comparisons were classified as ‘same species’ (distance ≤ threshold) or ‘different species’ (distance > threshold). Classification performance was assessed using overall accuracy, defined as the proportion of correctly classified pairwise comparisons.

Barcoding efficiency (BE) was defined as the maximum classification accuracy achieved at OT and thus represented the proportion of correctly classified pairwise comparisons under the best‐performing threshold for a given dataset.

For visualisation, distributions of intraspecific and interspecific K2P distances were plotted as boxplots, with optimal threshold values indicated as horizontal reference lines. Distances were expressed as percentage K2P genetic distance and displayed on a common scale to facilitate comparison among datasets.

All distance calculations were performed using a custom Python implementation of the K2P model. All analyses were conducted in Python 3 using custom scripts based on Biopython, NumPy, Pandas and Matplotlib.

### 2.9 Genetic Distances

Pairwise K2P genetic distances were calculated among BINs. All the analyses were conducted in Python 3 using the custom scripts detailed above. Pairwise distances were computed directly from aligned nucleotide sequences, with positions containing gaps or ambiguous bases (e.g., ‘N’) excluded on a pairwise basis. For each sequence pair, proportions of transitions (P) and transversions (Q) were estimated and K2P distances were calculated according to the standard formulation.

Distance matrices were subsequently converted to percentage values (0%–100%) for visualisation.

To facilitate interpretation of genetic structure, distance matrices were reordered using hierarchical clustering (average linkage) based on K2P distances. The resulting ordering was applied simultaneously to rows and columns to emphasise clustering patterns.

### 2.10 Intraspecific and Interspecific K2P Distance Calculation

Intraspecific mean and maximum K2P distances were calculated in the BOLD Workbench: Sequence Analysis → Distance Summary (Distance Model: Kimura 2 Parameter, Marker: COI‐5P‐Cytochrome Oxidase Subunit 1 5′ Region (7), Align Sequences*: MUSCLE (Edgar 2004), Apply Filters: Sequence Length ≥ 200 bp, Ambiguous Base/Gap Handling: Pairwise Deletion).

### 2.11 Maximum Likelihood Phylogenetic Analysis

Phylogenetic relationships among Diplopoda specimens were inferred using maximum likelihood (ML) as implemented in RAxML‐NG v1.x (Kozlov et al. 2019). The analysis was based on a multiple‐sequence alignment of COI, comprising 78 terminals and 675 base pairs.

To reduce redundancy, a single representative sequence per BIN was selected. The alignment was generated using MAFFT with the L‐INS‐i algorithm (Katoh and Standley 2013) and inspected prior to the analysis.

The substitution model was also specified in RAxML‐NG, as GTR + FO + G4m, corresponding to a general time‐reversible model with empirical base frequencies and gamma‐distributed rate heterogeneity across sites.

Tree inference was performed using the standard ML search algorithm. Branch support was assessed with 1000 non‐parametric bootstrap replicates, and support values were mapped onto the best‐scoring ML tree using the Felsenstein bootstrap proportion (FBP).

All analyses were conducted on a local workstation (Apple M1 architecture). The resulting phylogenetic tree was visualised and annotated using iTOL v6 Letunic and Bork (Letunic and Bork 2024).

### 2.12 Neighbour‐Joining (Supporting Information)

A Neighbour‐Joining (NJ) tree (Saitou and Nei 1987) was constructed for all COI sequences generated within this study, based on K2P distances. Node support was assessed using bootstrap resampling with 500 replicates (Felsenstein 1985), as implemented in MEGA XII (Kumar et al. 2024). A comprehensive Neighbour‐Joining tree including all COI sequences obtained in this study is presented in the Appendix S1.

### 2.13 Phylogenetic Network Analysis

Phylogenetic networks were constructed for selected genera to explore patterns of genetic divergence and potential conflict among sequences. Analyses were performed using SplitsTree 6, applying the NeighborNet algorithm under default settings (Huson and Bryant 2024). Apart from polish sequences, published sequences from other parts of Europe were used for comparison (Spelda et al. 2011; Wesener 2012; Wilbrandt et al. 2015) as well as other european resources publicly available in BOLD.

### 2.14 Distribution Maps

The maps showing the distribution of the studied species were created using QGIS 3.44.6 (Solothurn) (https://doi.org/10.5281/zenodo.6139224). The topographic basemap used in all maps presented in the figures was sourced from the terrestris Topographic WMS (https://ows.terrestris.de/osm/service).

## 3 Results

### 3.1 Species and BIN Diversity, Barcode Library Completeness

**FIGURE 3:**
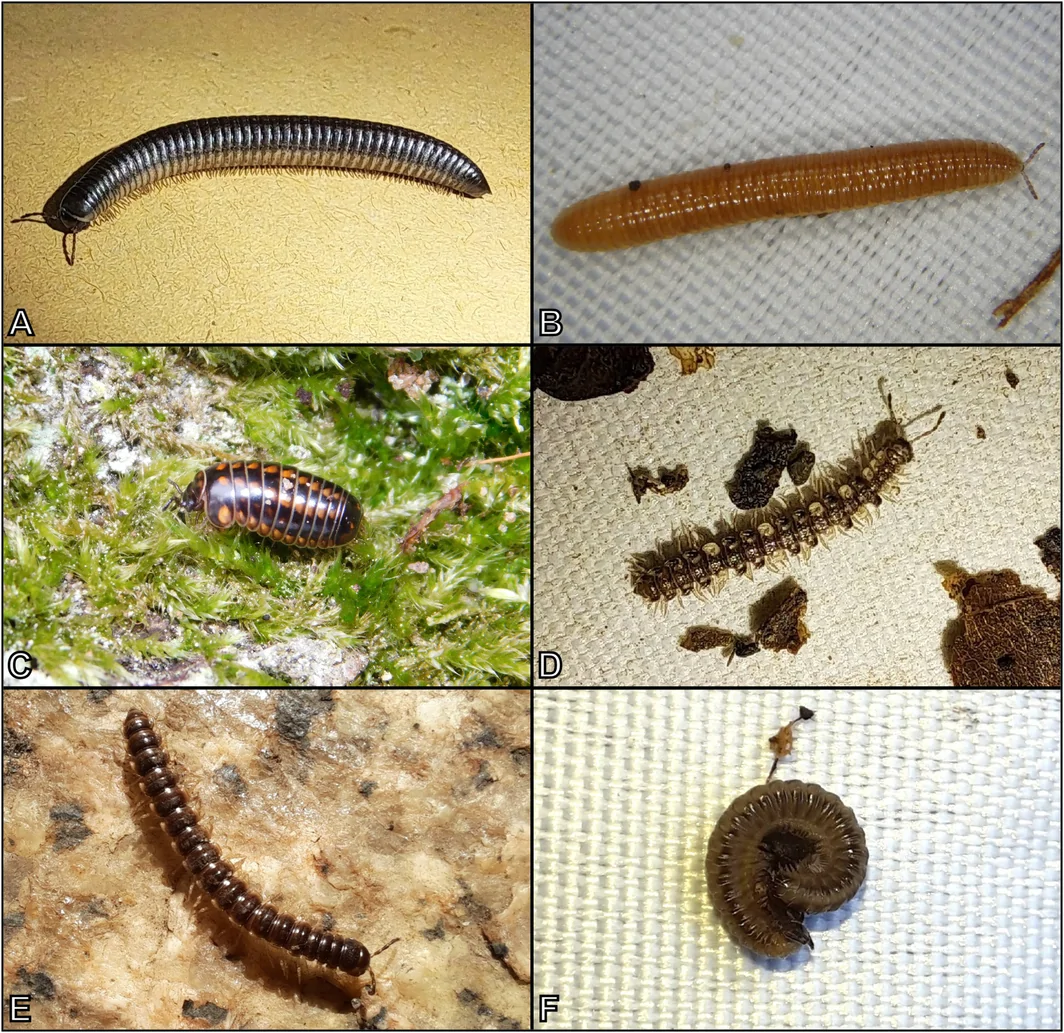
Examples of some polish diplopods for which COI barcodes are now available. *Ommatoiulus vilnensis* (A), *Polyzonium germanicum* (B), *Glomeris hexasticha* (C), *Polydesmus polonicus* (D), *Oxidus gracilis* (E), *Craspedosoma rawlinsii* (F).

In total, our dataset contains records of 976 specimens of 65 species occurring in Poland. Of that, 58 species were successfully barcoded (Figure 3), resulting in 465 COI sequences. The sequences were partitioned into 77 BINs, of which 28 are unique to BOLD. The number of BINs per species ranged from 1 to 4. The species with the highest number of BINs were *Glomeris tetrasticha* Brandt, 1833 and *Leptoiulus proximus* (Némec 1896). Additionally, alongside the molecular results, we provide an updated checklist of Polish millipede species (Appendix S2) and other important faunistic records. *Melogona broelemanni* (Verhoeff 1897b) is a species new to the Polish fauna. One adult male and two juveniles were found under a stone in a garden in Zakopane in November 2024. There are also additional records of two species relatively recently recorded from Poland—*Chondrodesmus riparius* and *Polydesmus angustus* (Bienias et al. 2021, 2025). In the case of *P. angustus*, two specimens were found in Golnice (51.34524 N, 15.58443 E), SW Poland, in a mixed forest near the road. Few new records of *C. riparius* have been reported on social media, from flowerpots: Augustów (53.8417 N, 22.9816 E), 9.11.2025, Wąbrzeźno (53.2789 N, 18.9471 E), 21.02.2026, Warsaw (52.2391 N, 20.9346 E), 10.09.2025. Additionally, we report a second record of *Ophiodesmus albonanus* (Latzel 1895) from Poland, and the first one from outside artificial conditions; one adult male was found under a stone in a strongly managed private garden in Gdynia (Baltic Sea coast).

### 3.2 ML Phylogeny and Barcode Gap Analysis

**FIGURE 4:**
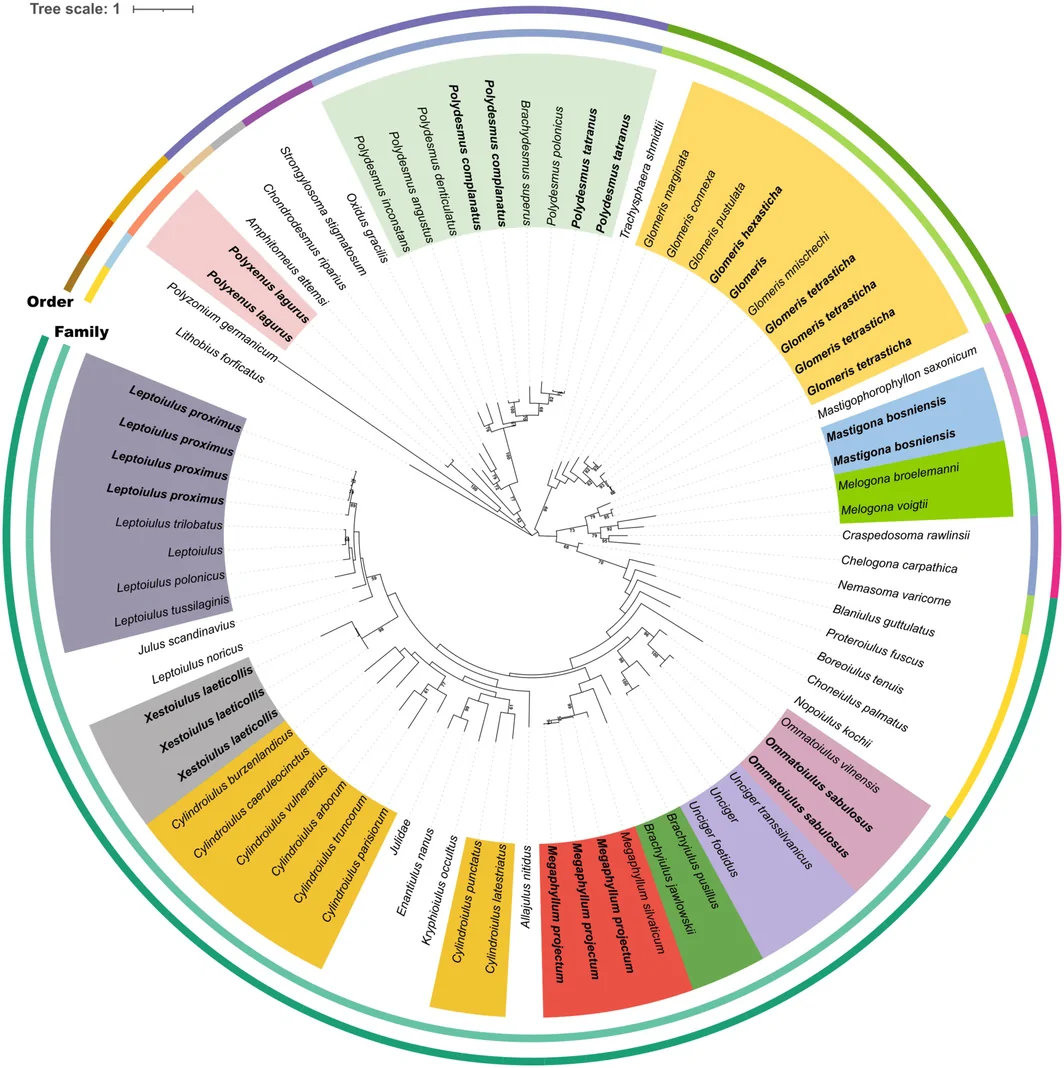
Maximum‐likelihood phylogenetic tree based on COI sequences of Polish Diplopoda. One representative sequence per BIN was included. Branch shading colours correspond to genera; single representatives of a genus in the tree have no shading. The inner ring indicates family‐level assignment, and the outer ring shows the respective orders.

The Maximum Likelihood phylogeny (Figure 4) recovered a clear clade structure corresponding to the major millipede orders and families represented in the dataset. All sequences of Polydesmidae clustered in a single, well‐supported lineage confirming the monophyly of the family. Representatives of Glomeridae likewise formed a distinct and well‐supported clade. Within Julidae, the ML tree revealed greater internal complexity, with a pronounced separation between the *Cylindroiulus* and *Leptoiulus* lineages, reflecting their deeper evolutionary divergence. Additional families, including Mastigophorophyllidae and Craspedosomatidae, also formed discrete, well‐supported clades that align with their current morphological classification. Nevertheless, Blaniulidae was recovered as paraphyletic. At the generic level, monophyly was not always consistently recovered as well. *Cylindroiulus* was retrieved as paraphyletic due to the inclusion of *Enantiulus* and *Kryphioiulus* within this clade. *Leptoiulus* was likewise not recovered as monophyletic, as *Julus scandinavius* was nested within *Leptoiulus*, suggesting the paraphyly of the latter genus.

The complete NJ tree, including all the Polish sequences together with their localities and BIN numbers, is provided in the supplement (Appendix S1).

**FIGURE 5:**
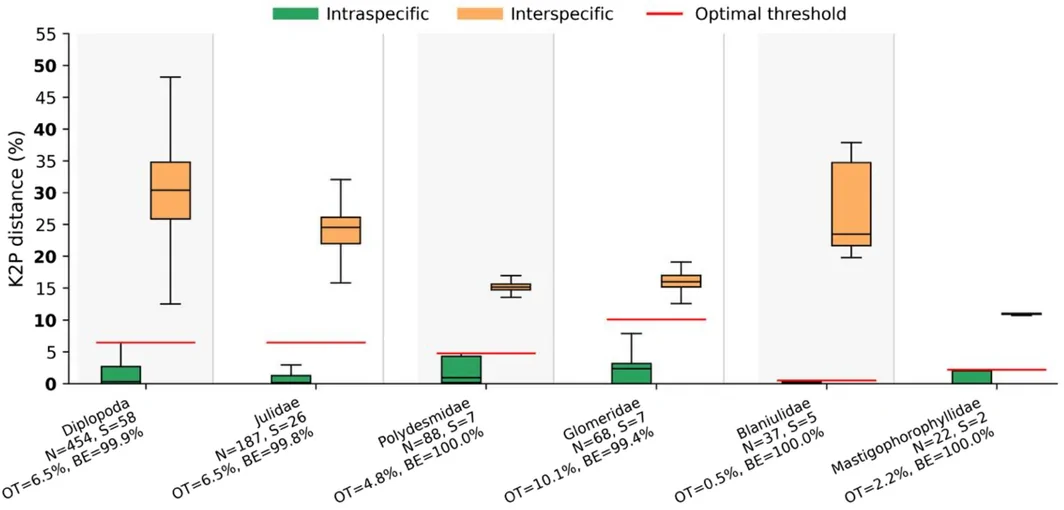
Species‐level barcode gap in Polish Diplopoda based on COI sequences. Boxplots show the distributions of intraspecific (green) and interspecific (orange) Kimura two‐parameter (K2P) genetic distances expressed as percentages for the complete Diplopoda dataset and the five most extensively sampled families. Intraspecific distances represent pairwise comparisons among specimens assigned to the same species, whereas interspecific distances represent comparisons among different species within the analysed dataset or family. Red horizontal lines indicate the optimal threshold (OT) maximising discrimination between intra‐ and interspecific comparisons. For each dataset, the number of analysed specimens (N), number of species (S), optimal threshold (OT) and barcoding efficiency (BE) are shown below the *x*‐axis. The *y*‐axis represents K2P genetic distance (%).

Analysis of species‐level COI barcode gaps revealed a clear separation between intraspecific and interspecific genetic distances across the Polish Diplopoda dataset (Figure 5). For the complete dataset comprising 454 sequences representing 58 species, the optimal threshold (OT) was 6.46%, with a barcoding efficiency (BE) of 99.93%.

Among the five most extensively sampled families, optimal thresholds ranged from 0.49% in Blaniulidae to 10.07% in Glomeridae. Barcoding efficiency reached 100% in Polydesmidae, Blaniulidae and Mastigophorophyllidae, while slightly lower values were observed in Julidae (99.82%) and Glomeridae (99.43%).

The highest maximum intraspecific divergence was observed in Julidae (17.19%), followed by Glomeridae (10.07%), whereas Blaniulidae exhibited very low intraspecific variation (maximum 0.49%). Minimum interspecific distances ranged from 4.64% in Glomeridae to 19.79% in Blaniulidae. Overall, the distributions of intraspecific and interspecific distances showed limited overlap, resulting in high species discrimination success across the analysed taxa (Figure 5).

### 3.3 Molecular Divergence and Phylogeographic Structure

**FIGURE 6:**
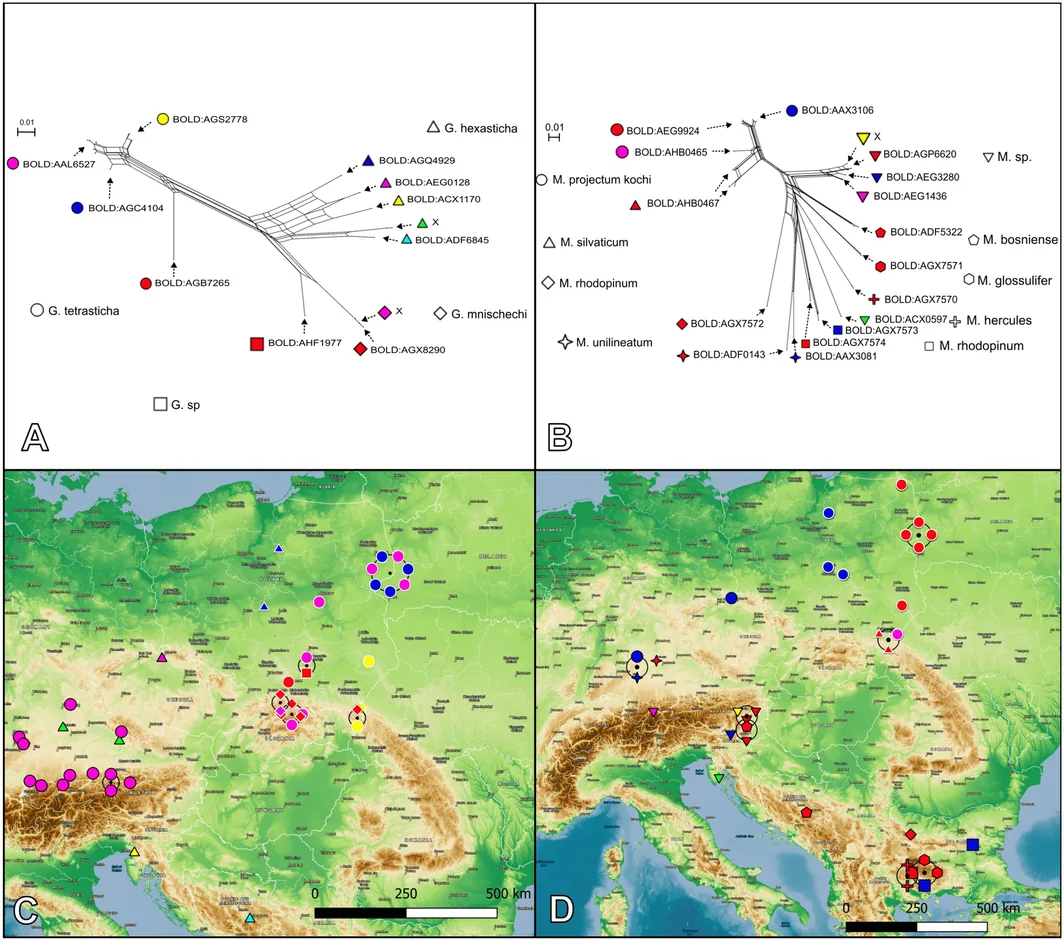
Phylogenetic network based on COI sequences illustrating genetic relationships among *Glomeris* (A) and *Megaphyllum* (B) species assigned to different BINs along with a map that reflects distribution of BINs in Europe for *Glomeris* (C) and *Megaphyllum* (D). Shape of symbol reflects species, while the colouration BIN type. Symbols with white outlines indicate Polish sequences obtained for the purpose of this study.

In some species, interesting phylogeographic patterns emerge, as seen in the genus *Glomeris*. For example, *G. tetrasticha* includes four BINs in Poland (Figure 6A,C); three of them are new to BOLD, since all other records, stemming exclusively from Germany, fall into a single BIN, BOLD:AAL6527. Analysis of individuals from Poland revealed the mean K2P distance reaching 2.47% (range: 0%–8.40%). These values align with the presence of multiple BINs and point to substantial genetic structure within the species. Notably, the structure within *G. tetrasticha* is driven by a single outlier specimen from Southern Poland (BOLD:AGB7265), markedly distinct (ca. 8%) from all the other individuals collected in the country. The remaining BINs were much less divergent. Most BINS are geographically isolated from each other; the only exception is an overlap of BOLD:AAL6527 and BOLD:AGC4104 in eastern Poland.

Another intriguing case is *Glomeris hexasticha* Brandt, 1833 (Figure 6A,C). Across Europe, this species shows exceptionally high intraspecific distances, with mean K2P values of 7.50% and a maximum of 10.75%, indicating the presence of deeply divergent mitochondrial lineages. Polish material fits into this broader pattern: although only two sequences are available, they display substantial intraspecific divergence and represent two highly divergent mitochondrial lineages, consistent with the pronounced phylogeographic structure observed across Europe.

The enigmatic Carpathian species *G. mnischechi* forms a single, genetically homogeneous cluster with very low intraspecific divergence (mean K2P values within species of 0.05%). In comparison to Polish *G. hexasticha*, it is separated by a substantial genetic gap: across all pairwise comparisons, the mean K2P divergence between two species reaches 11.91%, with a maximum of 12.64%. Intriguingly, the BIN BOLD:AHF:1977, being the closest relative of *G. mnischechi* and divergent from it by 4.95%, represents morphologically distinct yet unknown, possibly new to science, species of *Glomeris*.

Similarly, another Carpathian species *Leptoiulus polonicus* Jawłowski 1930 shows clear genetic separation from the Polish material of *Leptoiulus trilobatus* Verhoeff, 1894, with a mean interspecific K2P distance of 8.83% (range 8.32%–9.93%).

In contrast to *Glomeris*, *Megaphyllum projectum* (Verhoeff 1894b) (Figure 6B,D) exhibits shallow mitochondrial divergence among Polish specimens but pronounced spatial segregation of BINs, consistent with limited dispersal and regionally structured populations. Across the Polish material, the mean intraspecific K2P distance was 0.99% (range: 0%–4.8%), indicating relatively modest mitochondrial differentiation. Despite this, three BINs were detected, each showing a distinct geographic pattern. BIN BOLD:AEG9924 occurred exclusively in eastern Poland and formed a cohesive regional cluster. BOLD:AAX3106 was distributed mainly in central‐eastern Poland; a third BIN, BOLD:AHB0465, was represented by a single specimen from the south‐east of Poland.

In the case of species that show very shallow mitochondrial divergence within Poland, such as *Enantiulus nanus* (Latzel 1884a), *Mastigona bosniensis* (Verhoeff 1897a), or *Trachysphaera schmidtii* Heller 1858, the situation seems more complex when viewed in a broader European context.

**FIGURE 7:**
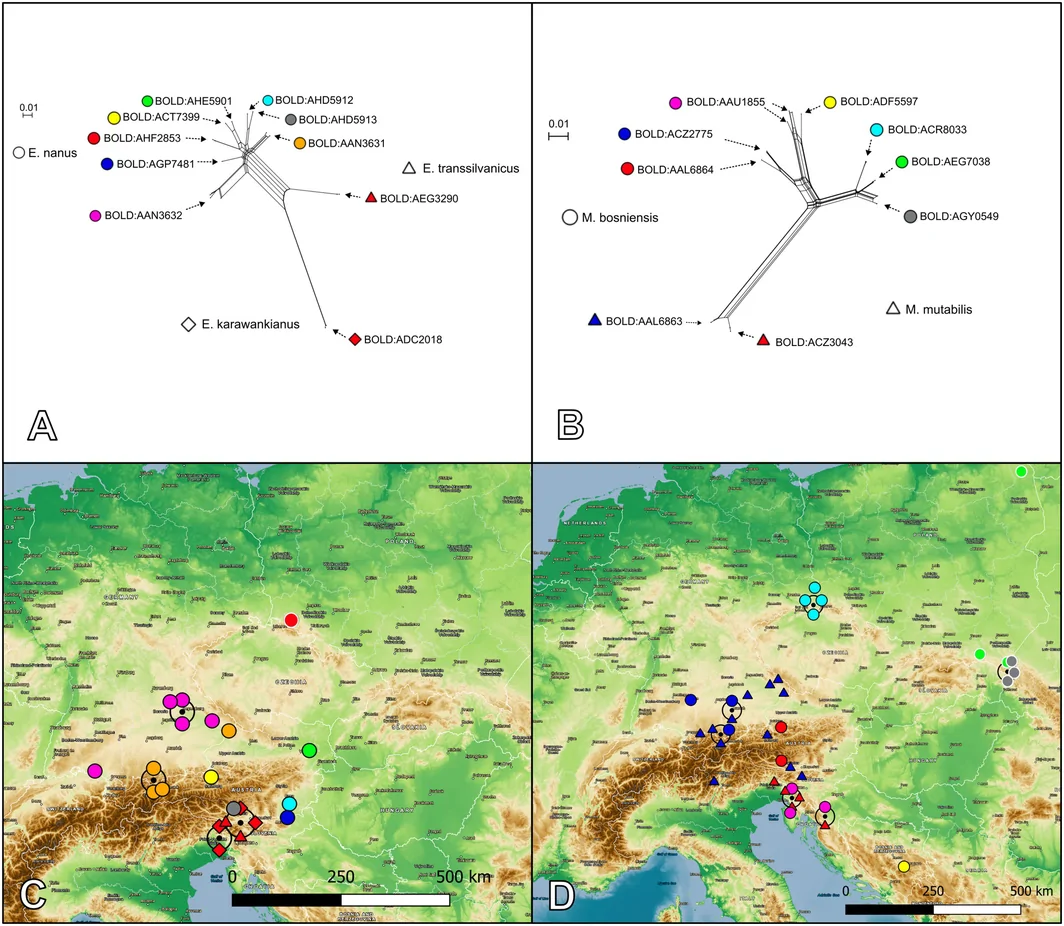
Phylogenetic network based on COI sequences illustrating genetic relationships among *Enantiulus* (A) and *Mastigona* (B) species assigned to different BINs along with a map that reflects distribution of BINs in Europe for *Enantiulus* (C) and *Mastigona* (D). Shape of symbol reflects species, while the colouration BIN type. Symbols with white outlines indicate polish sequences obtained for the purpose of this study.

In *Enantiulus nanus* (Figure 7A,C), Polish specimens showed no detectable mitochondrial divergence and were all assigned to a single BIN (BOLD:AHF2853), yet the broader European dataset revealed substantial intraspecific structuring. Across Europe, the intraspecific COI distances ranged from 5.96% to 9.34%, indicating the presence of several deeply divergent mitochondrial lineages within *E. nanus* that are not represented in the Polish material. However, given that our barcoding data for this species are restricted to a few specimens from the southwestern part of Poland, more comprehensive sampling across the country is necessary to clarify whether the population is genuinely uniform—reflecting a single glacial refugia origin—or if further mitochondrial lineages remain undetected.

In *Mastigona bosniensis* (Figure 7B,D), the individuals from Poland showed only shallow mitochondrial divergence, with two BINs occurring in the eastern part of the Polish Carpathians, and COI distances ranging from 0% to 2.17% (mean 1.06%). This pattern suggests limited local structuring without evidence of deep lineage separation. In contrast, the broader European dataset showed substantially higher intraspecific divergence, with COI distances averaging 4.16% and reaching 7.92%. These values indicate the presence of several well‐differentiated mitochondrial lineages across the species' range but not present in Poland.

**FIGURE 8:**
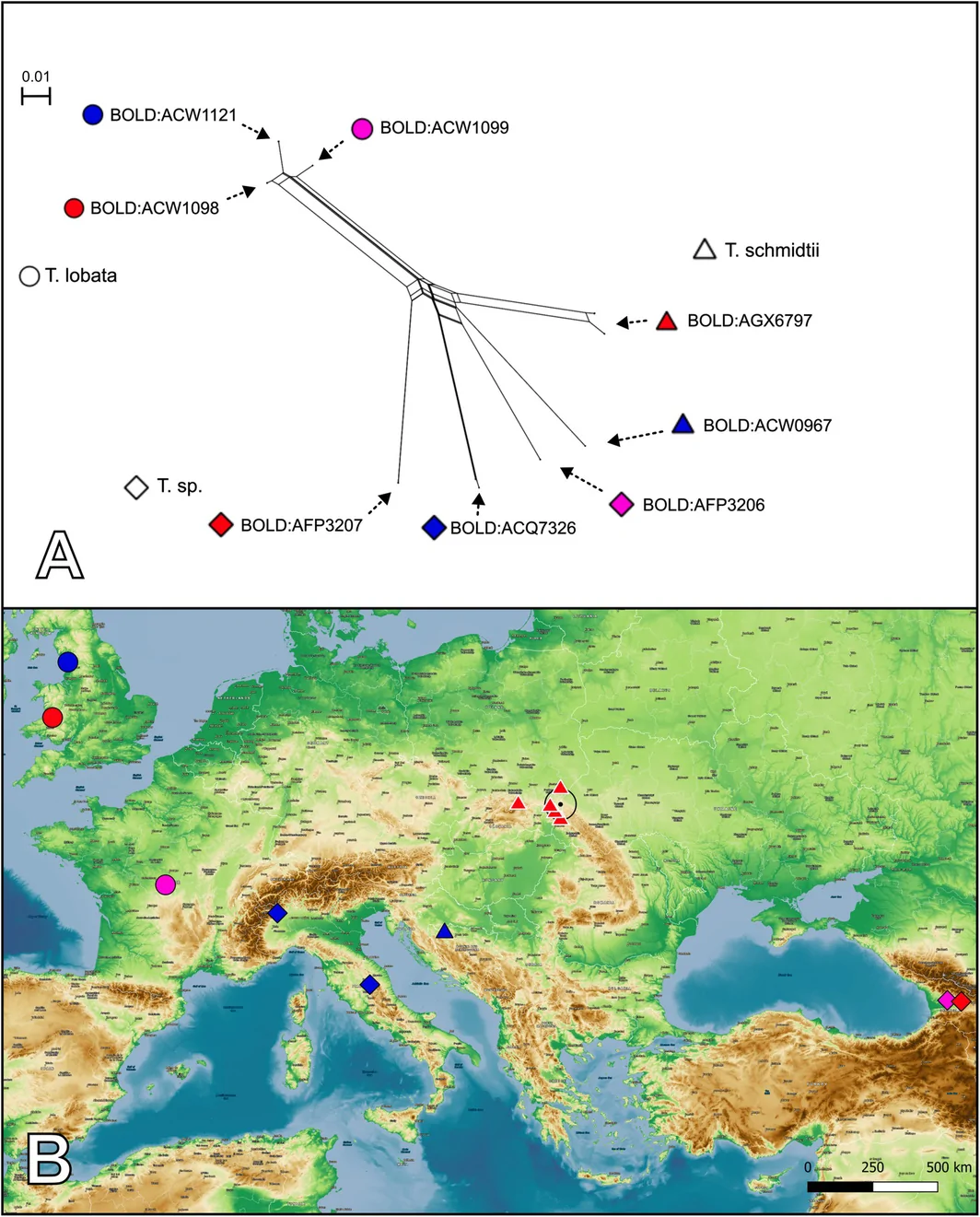
Phylogenetic network based on COI sequences illustrating genetic relationships among *Trachysphaera* (A) species assigned to different BINs along with a map that reflects distribution of BINs in Europe (B). Shape of symbol reflects species, while the colouration BIN type. Symbols with white outlines indicate polish sequences obtained for the purpose of this study.

In the case of *Trachysphaera schmidtii* (Figure 8A,B), all Polish individuals belonged to a single BIN (BOLD:AGX6797). When compared with the only publicly available foreign sequence (from Croatia), COI distances reached 14.01%, indicating a potentially substantial divergence between the Polish lineage and the Croatian specimen.

## 4 Discussion

Our study confirms the usefulness and underlines the importance of national efforts in generating reliable DNA barcoding reference libraries (Kaitetzidou et al. 2026), and the need for developing such a library for the identification of Polish diplopods. So far, 86 species of Diplopoda have been recorded from Poland in the checklist by Wytwer (2008). In recent years, several new millipede species have been reported (Tajovský and Wytwer 2010; Jastrzębski 2012; Bienias et al. 2021, 2025, 2026). A subsequent checklist (Wytwer and Bienias 2022), being a chapter of the Digital Catalogue of Biodiversity of Poland available through GBIF, serves as a publicly accessible data resource that is being recurrently updated. In the present study, we provide an updated and fully verified checklist of 93 species, including one species newly recorded for the Polish fauna (Appendix S2). Furthermore, until now the DNA barcode data for Polish diplopods was scarce; only three species had DNA barcodes (Bienias et al. 2021, 2025, 2026). Our study updated this to 58 species, which is 62% of Polish diplopod fauna.

### 4.1 Changes in the Polish Millipede Fauna and Range Expansions

Regarding the new record for the Polish fauna, Melogona broelemanni was also recently reported from Germany (Decker et al. 2025), which may suggest that its distribution is currently expanding in Europe. The mysterious julid specimen that was found in Sopot, morphologically, is clearly a new record for the Polish fauna. However, since it is a female, it could not be identified to species level. Morphologically, it is similar to *Enantiulus armatus* (Ribaut 1909), a species with Atlantic distribution that occurs in France and Great Britain (Kime and Enghoff 2017). Although we could successfully sequence the DNA‐barcode of this specimen, DNA barcoding could not help to assign a species name, since matching references are missing from BOLD. Therefore, we currently leave this specimen unidentified until new material or comparative data becomes available. Few species seem to expand their range in Poland. Records of *Leptoiulus trilobatus* were historically limited to upland and mountainous areas of southern Poland (Stojałowska 1961; Dziadosz 1966). Our records from Wolbórka Nature Reserve (51.628 N, 19.526E) and Lipickie Błota Nature and Landscape Complex (51.847713 N, 18.46953E) indicate a possible expansion of the range of this species into the lowlands of central parts of Poland. *Melogona voigtii* (Verhoeff 1899) was until now known only from a few localities in northern and south‐western Poland: Zgorzelec, Elbląg and Szklarska Poręba. (Stojałowska 1961; Bielak‐Oleksy and Jaśkiewicz 1977). Our records of multiple specimens from Bytom and Warsaw suggest that the distribution of this species may be largely underestimated and it might be present in synanthropic habitats in other areas of the country. The earliest and only confirmed record of *O. albonanus* in Poland dates back to 1982, when a solitary male was discovered in a greenhouse at the Botanical Garden of the University of Warsaw (Jędryczkowski 1982). In neighbouring Germany, occurrences of *O. albonanus* are considerably more numerous, mostly in synanthropic habitats, although the species is still regarded as uncommon even there (Kime and Enghoff 2011; Voigtländer and Decker 2014; Decker et al. 2025).

### 4.2 Molecular Structure of Diplopoda Species and Phylogeographic Patterns in Poland

The COI phylogeny recovered most sampled families and orders as distinct and well‐supported lineages, indicating that the barcode marker retains a strong phylogenetic signal at shallow and intermediate taxonomic levels. However, relationships among major diplopod lineages differed from those recovered in recent phylogenomic analyses based on ultraconserved elements (UCEs), which provide substantially higher resolution of deep evolutionary relationships (Shapiro et al. 2026; Vasquez‐Valverde et al. 2026). Therefore, the higher‐level topology recovered here should be interpreted cautiously and primarily as a visualisation of barcode diversity rather than a robust hypothesis of ordinal‐level relationships.

The levels of intraspecific COI divergence observed in the present study were substantially higher than those reported by Spelda et al. (2011) for Bavarian Diplopoda, who found a mean intraspecific distance of 0.82% and a maximum of 6.61% in *Glomeridella bitaeniata*. In contrast, in our dataset, the maximum intraspecific divergence reached 17.19% in Julidae and 10.07% in Glomeridae. Such elevated values may reflect the presence of geographically structured lineages, overlooked cryptic diversity, or taxonomic inconsistencies within currently recognised species. Despite this increased intraspecific variation, minimum interspecific distances remained higher than maximum intraspecific distances in most families, resulting in a well defined barcode gap and high species discrimination success.

It is clear that COI variability in Polish millipedes is substantial in several lineages and warrants further investigation, ideally integrating additional molecular markers and morphological data. This is particularly evident in species that show substantial COI divergence either within Poland or across Europe, as well as in taxa where multiple mitochondrial lineages occur allopatrically, such as *Glomeris*, *Megaphyllum*, *Enantiulus*, *Mastigona* or *Trachysphaera*. Up till now the interpretation of these patterns is constrained by limited geographic sampling in several species. In *M. projectum*, the presence of BIN BOLD:AAX3106 in Germany may indicate a western origin of this lineage, but this cannot be verified without broader sampling. Likewise, the single Polish specimen representing BIN BOLD:AHB0465 could reflect a localised lineage, though its stability and range remain unknown. In *Enantiulus nanus*, all Polish specimens belong to BIN BOLD:AHF2853, yet the species shows deep mitochondrial structuring at the European scale, making additional sampling necessary to determine whether the homogeneity of Polish populations reflects a single postglacial recolonisation. In *Mastigona bosniensis*, the two BINs detected in the Polish Carpathians show only shallow mitochondrial divergence, whereas the species displays much deeper divergence across Europe, suggesting that the Polish BINs represent only a small portion of its continental diversity. For *Trachysphaera schmidtii*, the deep divergence between the Polish BIN BOLD:AGX6797 and the single Croatian sequence must be interpreted cautiously due to extremely sparse foreign data. However, as more species and specimens are barcoded, this pattern may change. The results also highlight how the status of well‐studied and common species in Poland, such as *G. tetrasticha*, may become more complex in light of new molecular evidence. These patterns also point to the possibility of cryptic diversity within several Polish diplopod species, particularly where deeply divergent mitochondrial lineages coexist under a single morphological species name. Furthermore, DNA barcoding can help clarify and confirm the identity of poorly known or taxonomically problematic species such as *G. mnischechi*. The value of DNA barcoding for studying pill millipedes is already well established. Building on this foundation, genetic analyses within *Glomeris* have revealed overlooked synonyms, exposed cryptic lineages and clarified several taxonomic uncertainties (Hoess and Scholl 1999, Wesener 2015, Wesener and Conrad 2016, Wesener 2018). *G. mnischechi* has traditionally been regarded as the only Glomerida endemic north of the Alps, yet its taxonomy has long been complicated by inconsistent early descriptions, numerous synonyms and its close similarity to *G. hexasticha* (Haľková et al. 2021). It presumably inhabits woodlands in the Tatra Mountains in Poland and Slovakia (Stojałowska 1961; Kime and Enghoff 2011). Although its status in the past has remained largely unresolved, our molecular data from the type region suggest that *G. mnischechi* may indeed represent a valid species (see Figure 6). It is also worth noting that all *G. mnischechi* specimens not only belong to a single BIN and form a uniform cluster separated from *G. hexasticha* (mean distance between species 9.55%), but were also morphologically identified to the same species by different specialists (Cezary Dziadosz and Hieronim Jawłowski, unpublished data from the collection of MIIZ PAN), which greatly reduces the chance of misidentification. Notably, the lineage represented by BIN BOLD:AHF1977, belonging to what is currently referred to as *Glomeris* sp., shows an unexpectedly close affinity to *G. mnischechi*, with a mean divergence of only 4.96%. This clustering with *G. mnischechi* may indicate a new, yet undescribed species closer to *G. mnischechi* than to *G. hexasticha*. A somewhat similar situation occurs in the north‐eastern Carpathian species *Leptoiulus polonicus* Jawłowski 1930. This rather poorly known species, which occurs in Poland and Ukraine, was in the past treated as a subspecies of *Leptoiulus trilobatus* (Stojałowska 1961). However, our results, based on specimens from Ukraine, indicate that it is clearly a separate species, with the mean K2P distance between it and *L. trilobatus* being approximately 9%. Another interesting case within *Leptoiulus* is *L. noricus* Verhoeff 1913. The two sequences we obtained from specimens in the MIIZ PAN collection fall into a single BIN, BOLD:AHB0671 (Figure 4). However, the taxonomic status of this species remains unclear. These specimens were originally identified by Dziadosz as *Leptoiulus simplex marcomannius* Verhoeff 1913 (unpublished data from the collection of MIIZ PAN). This subspecies (or species, when treated as *Leptoiulus marcomannius* Verhoeff 1913) was, until very recently, considered a synonym of *L. noricus* (Kime and Enghoff 2017) and was treated as such in the Polish diplopod checklist of Wytwer (2008). Recent studies, however, suggest a more complex situation. *L. marcomannius* may indeed represent a distinct species that originated through the stabilisation of an Alpine hybrid population between *Leptoiulus simple*x Verhoeff, 1894a and *L. noricus*. (Decker et al. 2026). Nevertheless, additional morphological and genetic data are required to clarify the status of this species group. Our sequences were identified in the BOLD ID Engine as closest to *L. noricus* specimens from Austria. Considering all of the above, and following the approach of the previous checklist (Wytwer 2008), we treat the specimens collected and identified by Dziadosz as *L. simplex marcomannius* as *L. noricus* for the purposes of this study, until the situation is resolved. In contrast to allopatric cases, some species exhibit multiple BINs that either lack any clear geographic provenance or occur in true sympatry. For example, in *Leptoiulus proximus*, four BINs are recorded from Poland (Figure 4), yet their spatial relationships remain unclear and all show only moderate intraspecific K2P divergence (mean 2.6%, max 4.6%), with no indication of coherent geographic structuring. A clearer case is provided by *Polyxenus lagurus* (Linnaeus 1758), where two BINs (Figure 4) display low inter‐BIN K2P distances (typically below 4%) (see Figure 4) and occur in exactly the same localities, demonstrating genuine sympatry. Together, these cases illustrate that the presence of multiple BINs does not necessarily reflect the presence of cryptic species but instead reflects shallow population genetic structure. Another argument for being cautious when interpreting COI results is the potential presence of *Wolbachia* infection, which is known to affect mtDNA polymorphism. *Wolbachia* is a maternally inherited endosymbiont capable of manipulating host reproduction (e.g., cytoplasmic incompatibility, male killing, feminization), thereby increasing the relative fitness of infected female lineages. Because both endosymbiont and mitochondria are transmitted strictly through the maternal line, the spread of the infection can drive a selective sweep of the associated mitochondrial haplotype, reducing mtDNA diversity and distorting phylogeographic or species‐level signals (Cariou et al. 2017). The first confirmed record in millipedes is relatively recent—from 2016 (Mock et al. 2016), consequently within myriapods it is known from only in a handful of cases. Similar effects can also be caused by *Rickettsia*, which has been found, for example, in *Polydesmus complanatus* (Linnaeus, 1761) (Li et al. 2019). Thus, the influence of such endosymbionts cannot be fully excluded in some instances.

## 5 Conclusions

Overall, our findings clearly demonstrate that the Polish diplopod fauna is undergoing noticeable and ongoing changes, reflected in shifting species distributions and the appearance of species newly recorded for the national fauna. At the same time, in light of newly generated molecular data, previously unrecognised genetic structure is becoming increasingly evident. These patterns underscore the need for continued monitoring of millipede diversity in Poland, particularly in the context of expanding, synanthropic, or taxonomically problematic species. DNA barcoding plays a central role in this process: it not only enables reliable species‐level identification but also reveals deeply divergent mitochondrial lineages, highlights cases of potential cryptic species and helps refine our understanding of species boundaries. As barcode reference libraries grow and additional molecular markers are incorporated, future studies will be able to resolve these patterns with even greater precision, ultimately providing a more complete picture of the evolutionary and biogeographic processes shaping the diplopod fauna of Poland. Another major contribution of this study is the updated checklist of Polish species, which provides a reliable framework for future research.

## Author Contributions

**Jakub Bienias:** conceptualization (lead), data curation (equal), formal analysis (equal), funding acquisition (lead), investigation (lead), methodology (equal), project administration (lead), writing – original draft (lead). **Łukasz Trębicki:** formal analysis (equal), methodology (equal), project administration (equal), supervision (equal), writing – original draft (equal). **Gabriela Karlik:** methodology (equal), resources (equal), writing – original draft (equal). **Michał Grabowski:** conceptualization (lead), project administration (lead), supervision (lead), writing – original draft (equal).

## Funding

The study was performed and partially funded within the Polish Barcode of Life (PoLBOL, polbol.uni.lodz.pl) initiative. The study was funded by the project ‘Diversity, biogeography and integrative taxonomy of myriapods in Poland’ (Förderkreis für Allgemeine Naturkunde (Biologie), Germany) (https://www.fan‐b.de) as well as BGE (Biodiversity Genomics Europe). BGE was funded by Horizon Europe (Grant no. 101059492) under the Biodiversity, Circular Economy and Environment call (REA.B.3), co‐funded by the Swiss State Secretariat for Education, Research and Innovation (SERI) under contract numbers 22.00173 and 24.00054 and by the UK Research and Innovation (UKRI) under the Department for Business, Energy and Industrial Strategy's Horizon Europe Guarantee Scheme.

## Ethics Statement

The authors have nothing to report.

## Consent

The authors have nothing to report.

## Conflicts of Interest

The authors declare no conflicts of interest.

## Supporting information


**Appendix S1:** Full Neighbour‐Joining tree of Polish Diplopoda species.

**Appendix S2:** Checklist of Polish Diplopoda species.

## Data Availability

The data that support the findings of this study are openly available in BOLD SYSTEMS at https://portal.boldsystems.org/recordset/DS‐PDBL.
