# Supplementary material for: First Taxonomically Curated DNA Barcode Reference Library for Polish Myriapods Fills an Important Knowledge Gap for Soil Invertebrates in Central Europe, Part I: Millipedes (Myriapoda: Diplopoda)
Source: Ecol Evol. 2026 Sep 3;16(9):e74268. doi: 10.1002/ece3.74268 (PMC13542578; doi:10.1002/ece3.74268)

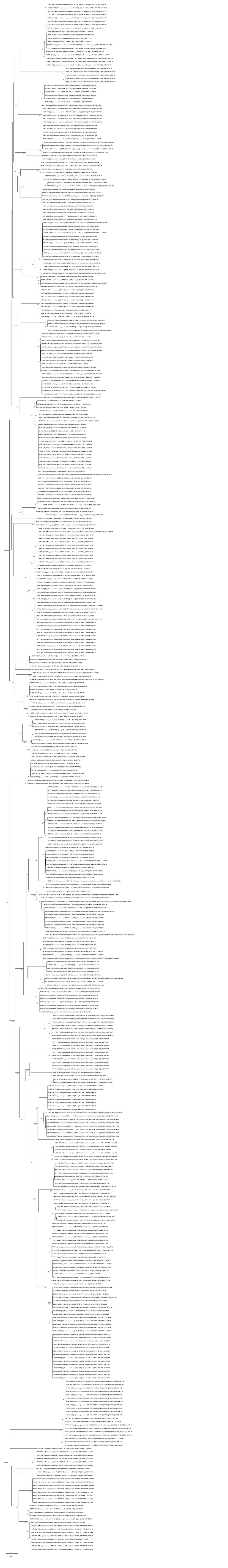

Supplement: Supplementary file 1 [file ECE3-16-e74268-s001.pdf]
