# Supplementary material for: First Taxonomically Curated DNA Barcode Reference Library for Polish Myriapods Fills an Important Knowledge Gap for Soil Invertebrates in Central Europe, Part I: Millipedes (Myriapoda: Diplopoda)
Source: Ecol Evol. 2026 Sep 3;16(9):e74268. doi: 10.1002/ece3.74268 (PMC13542578; doi:10.1002/ece3.74268)
Supplement: Supplementary file 2 [file ECE3-16-e74268-s002.docx]

**Diplopoda of Poland – Checklist**

**Chordeumatida**

Chordeumatidae

1. *Chordeuma sylvestre* C. L. Koch, 1847
2. *Melogona broelemanni* (Verhoeff, 1897)* c **BOLD:AAM0667**
3. *Melogona voigti* (Verhoeff, 1899)* c **BOLD:AAL6878**
4. *Mycogona germanica* (Verhoeff, 1892) p

Craspedosomatidae

5. *Chelogona carpathica* (Latzel, 1882)* c, p **BOLD:AGW6022**
6. *Craspedosoma rawlinsii* Leach, 1816* c **BOLD:AAM3827**
7. *Ochogona caroli* (Rothenbuhler, 1900) p
8. *Ochogona jankowskii* (Jawłowski, 1938)

Haaseidae

9. *Haasea flavescens* (Latzel, 1884)
10. *Hylebainosoma tatranum* Verhoeff, 1900

Mastigophorophyllidae

11. *Haploporatia eremita* (Verhoeff, 1909) p
12. *Karpatophyllon polinskii* Jawłowski 1928 p
13. *Mastigona bosniensis* (Verhoeff, 1887)* c, p **BOLD:AEG7038, BOLD:AGY0549**
14. *Mastigophorophyllon cirriferum* Verhoeff, 1889
15. *Mastigophorophyllon saxonicum* Verhoeff, 1910* c **BOLD:AGB4263**
16. *Mastigophorophyllon serrulatum* Attems 1926

**Glomerida**

Glomeridae

17. *Glomeris connexa* C. L. Koch, 1847* p **BOLD:AAL6526**
18. *Glomeris hexasticha* Brandt, 1833* c **BOLD:AGQ4929**
19. *Glomeris klugii* Brandt, 1833
20. *Glomeris marginata* (Villers, 1789)* c **BOLD:AAL6528**
21. *Glomeris mnischei* Nowicki, 1870* c, p **BOLD:AGX8290**
22. *Glomeris pustulata* (Fabricius, 1781)* c, p **BOLD:AAL6928**
23. *Glomeris tetrasticha* Brandt, 1833* c **BOLD:AAL6527, BOLD:AGB7265, BOLD:AGC4104, BOLD:AGS2778**
24. *Trachysphaera costata* (Waga, 1858)
25. *Trachysphaera gibbula* (Latzel, 1884)
26. *Trachysphaera schmidti* Heller, 1858* c, p **BOLD:AGX6797**

**Julida**

Blaniulidae

27. *Archiboreoiulus pallidus* (Brade-Briks, 1920)
28. *Blaniulus guttulatus* (Fabricius, 1798)* c **BOLD:AEG2977**
29. *Boreoiulus tenuis* (Bigler, 1913)* p **BOLD:AAO1366**
30. *Choneiulus palmatus* (Nemec, 1895)* c **BOLD:AAO0683**
31. *Cibiniulus phlepsi* (Verhoeff, 1897)
32. *Nopoiulus kochii* (Gervais, 1847)* c **BOLD:AEG9412**
33. *Proteroiulus fuscus* (Am Stein, 1857)* c **BOLD:AAN4174**

Julidae

34. *Allajulus nitidus* (Verhoeff, 1881)* c **BOLD:AAN3958**
35. *Brachyiulus bagnalli* (Brolemann, 1924)
36. *Brachyiulus jawłowskii* Lohmander, 1928* c **BOLD:AHF8338**
37. *Brachyiulus pusillus* (Leach, 1814)* c **BOLD:AAM7944**
38. *Cylindroiulus arborum* Verhoeff, 1928* c	**BOLD:AEG4394**
39. *Cylindroiulus britannicus* (Verhoeff, 1891)
40. *Cylindroiulus burzenlandicus* Verhoeff, 1907* c **BOLD:AGC0092**
41. *Cylindroiulus caeruleocinctus* (Wood, 1864)* c **BOLD:AAH7472**
42. *Cylindroiulus horvathi* (Verhoeff, 1897)
43. *Cylindroiulus latestriatus* (Curtis, 1845)* c **BOLD:AAM7943**
44. *Cylindroiulus parisiorum* (Brolemann et Verhoeff, 1896)* c **BOLD:ADF5352**
45. *Cylindroiulus punctatus* (Leach, 1815)* c **BOLD:AAI9434**
46. *Cylindroiulus truncorum* (Silvestri, 1896)* c **BOLD:ACX1038**
47. *Cylindroiulus vulnerarius* (Berlese, 1888) * c **BOLD:ACY0542**
48. *Enantiulus nanus* (Latzel, 1884)* c **BOLD:AHF2853**
49. *Enantiulus* *tatranus* (Verhoeff, 1907)
50. *Enantiulus transsilvanicus* (Verhoeff, 1899)
51. *Julus scandinavicus* Latzel, 1884* c **BOLD:AAH7469**
52. *Julus terrestris* Linnaeus, 1758
53. *Kryphioiulus occultus* (C. L. Koch, 1887)* c **BOLD:AEG2710**
54. *Leptoiulus baconyensis* (Verhoeff, 1889)
55. *Leptoiulus cibdellus* (Chamberlin, 1921) p
56. *Leptoiulus korongisius* (Attems 1904)
57. *Leptoiulus liptauensis* (Verhoeff, 1899)
58. *Leptoiulus noricus* Verhoeff, 1913* p **BOLD:AHB0671**
59. *Leptoiulus polonicus* Jawłowski, 1930* p	**BOLD:AHB0670**
60. *Leptoiulus proximus* (Nemec, 1896)* c, p **BOLD:AAX5694, BOLD:AEG8368, BOLD:AHA7063, BOLD:AHB0466**
61. *Leptoiulus trilobatus* Verhoeff, 1894* c, p **BOLD:ACR7984**
62. *Leptoiulus tussilaginis* (Verhoeff, 1907)* p **BOLD:AHB0669**
63. *Megaphyllum projectum* Verhoeff, 1894* c, p **BOLD:AAX3106, BOLD:AEG9924, BOLD:AHB0465**
64. *Megaphyllum silvaticum* (Verhoeff, 1898)* p **BOLD:AHB0467**
65. *Megaphyllum sjaelandicum* (Meinert, 1868)
66. *Megaphyllum unilineatum* (C. L. Koch, 1838)
67. *Ommatoiulus sabulosus* (Linnaeus, 1758)* p **BOLD:AAV3155, BOLD:ACS9855**
68. *Ommatoiulus vilinensis* (Jawłowski, 1925)* c, p **BOLD:AEG8881**
69. *Ophyiulus pilosus* (Newport, 1843) c
70. *Unciger foetidus* (C. L. Koch, 1847)* c **BOLD:AAO0528**
71. *Unciger transsilvanicus* (Verhoeff, 1899)* c **BOLD:AHG1705**
72. *Xestoiulus carpathicus* (Verhoeff, 1907)
73. *Xestoiulus laeticollis* (Porat, 1889)* c, p **BOLD:AGB1357,	BOLD:AHE6387**

Nemasomatidae

74. *Nemasoma varicorne* C. L. Koch, 1847* c **BOLD:AAN4181**

**Polydesmida**

Chelodesmidae

75. *Chondrodesmus riparius* Carl, 1914* c **BOLD:AEF9296**

Macrosternodesmidae

76. *Ophiodesmus albonanus* (Latzel, 1895) c

Oniscodesmidae

77. *Amphitomeus attemsi* (Schubart, 1934)* c **BOLD:ACT5943**

Paradoxosomatidae

78. *Helicorthomorpha holstii* (Pocock, 1895) c (Bienias 2026 in press)
79. *Oxidus gracilis* (C. L. Koch, 1847)* c **BOLD:AAG5070**
80. *Strongylosoma stigmatosum* (Eichwald, 1830)* c **BOLD:AAN9265**

Polydesmidae

81. *Brachydesmus superus* Latzel, 1884* c **BOLD:AAZ0391**
82. *Polydesmus angustus* Latzel, 1884* c **BOLD:AAF1373**
83. *Polydesmus burzenlandicus* Verhoeff, 1925
84. *Polydesmus complanatus* (Linnaeus, 1761)* c **BOLD:ACT7735, BOLD:ACZ3283**
85. *Polydesmus denticulatus* C. L. Koch, 1847* c **BOLD:AAN4290**
86. *Polydesmus inconstans* Latzel, 1884* c	**BOLD:AAM7947**
87. *Polydesmus komareki* Gulicka, 1962
88. *Polydesmus montanus* Daday 1889
89. *Polydesmus polonicus* Latzel, 1884* c **BOLD:AGR6626**
90. *Polydesmus tatranus* Latzel, 1882* p **BOLD:AHA8838**

Polyxenida

Polyxenidae

91. *Polyxenus lagurus* (Linnaeus, 1758)* c **BOLD:AAN3574, BOLD:AED4609**

**Polyzoniida**

Polyzoniidae

92. Polyzonium eburneum Verhoeff, 1907
93. *Polyzonium germanicum* Brandt, 1837* c **BOLD:AAY3544**

**Legend:**

* - species with DNA barcode

c - species in the collection of Department of Invertebrate Zoology and Hydrobiology, University of Lodz.

p - species in the collection of MIIZ PAN.
